# Microencapsulated Phase Change Material Suspension for Cold Start of PEMFC

**DOI:** 10.3390/ma14061514

**Published:** 2021-03-19

**Authors:** Sitong Chen, Shubo Wang, Xueke Wang, Weiwei Li, Baorui Liang, Tong Zhu, Xiaofeng Xie

**Affiliations:** 1 School of Mechanical Engineering and Automation, Northeastern University, Shenyang 110819, China; 1510112@stu.neu.edu.cn (S.C.); 1810136@stu.neu.edu.cn (B.L.); 2 Institute of Nuclear and New Energy Technology, Tsinghua University, Beijing 100084, China; wangshubo@mail.tsinghua.edu.cn (S.W.); willar@mail.tsinghua.edu.cn (W.L.); 3 Beijing Institute of Space Launch Technology, Beijing 100076, China; 1810135@stu.neu.edu.cn

**Keywords:** proton exchange membrane fuel cell, microencapsulated phase change suspension, cold start, thermophysical properties, thermal management

## Abstract

We added microencapsulated phase change materials (MPCMs) into the homemade antifreeze fluid to take advantage of the latent heat of phase change materials, and explored the possibility of solving the cold start problem of proton exchange membrane fuel cells (PEMFC) with variable specific heat capacity antifreeze. The physical and chemical properties of the MPCMs and their suspensions were tested, and a PEMFC platform for cold start with a thermal management system was established to compare the exothermic performance of MPCS and commercial antifreeze fluid. According to the output voltage, temperature and polarization curves before and after cold start, the MPCMs has a stronger heat transfer capacity than the commercial antifreeze fluid, and the addition of MPCMs can transform the latent heat generated during the phase transition into apparent specific heat capacity, leading to a better solution to the problem of PEMFC cold start.

## 1. Introduction

To build a competitive, secure and sustainable clean-energy economy, the proton exchange membrane fuel cells (PEMFCs), which convert hydrogen fuel directly into electricity without combustion, have become an alternative solution to energy depletion and environmental issues [1,2,3,4]. Due to PEMFC’s numerous strengths, including low operating temperatures, zero-emission, rapid response, high efficiency and power density, its commercialization process is vigorously promoted worldwide [5,6,7]. Nevertheless, there are still some obstacles to restrict its large-scale application; one of them is cold start failure, which is one of the thermal management issues of PEMFC. 

When PEMFCs are operated in cold winter conditions, the liquid water produced by the electrochemical reaction inside PEMFCs will freeze, the volume of which expands by 9%, leading to some irreversible damage caused by mechanical stresses like ruptures and pinholes in the membrane electrode assembly (MEA) and the interface delamination of the catalyst layer (CL) from the membrane or the gas diffusion layer (GDL) from the CL [8,9,10,11]. While the accumulated water exceeds the void volume of the porous layers (CL, GDL), the formed ice will block the catalyst layer and reduce the electrochemical active surface area [12,13]. Therefore, the voltage sag and cold start failure will inevitably occur. Tajiri et al. [14] investigated the effects of proton exchange membrane’s properties on cold start performance of a PEMFC. They concluded that the optimal performance was obtained with the lowest initial water content, because more product water was absorbed by the membrane, leaving only a small amount of water in the catalyst layer. Ishikawa et al. [15] showed that the critical cluster diameter, which was the maximum radius at which a supercooled state of the water within the porous layers could be maintained, was strongly dependent on temperature, the hydrophobicity and pore size of the CL material. Ko et al. [16] reported the effects of key CL design parameters on the cold start behavior of a PEMFC, such as the ionomer fraction and weight ratio of Pt to carbon support in the cathode CL. The calculated results confirmed that these two design parameters provided control of the ice storage capacity and water absorption potential of the cathode CL.

In order to improve the startup performance of PEMFCs and achieve rapid cold start, on the one hand, enhancing the water storage capacity in terms of design parameters of the CL materials is beneficial. On the other hand, it is necessary to minimize the mass of ice forming. Purging the residual water that remains in the PEMFC system by effective methods at shutdown is the first option [17,18,19,20]. In addition, the temperature of the PEMFC stack needs to reach the freezing point of water as soon as possible. Reviewing the published literature on quick heating research, the solutions can be classified into external heating and internal heating according to the heat generated from an external heating source or within the stack. The internal heating mainly warms up the stack by utilizing the reactant starvation [21,22], the load control of start mode (potentiostatic startup or galvanostatic startup, etc.) [23,24] and the hydrogen-oxygen reaction which adds some cathode reactant into the anode inlet [25,26]. As for external heating, one way it heats is by using the heat generated by hydrogen combustion in catalytic burners [27,28,29], and the other way requires connecting batteries to provide additional potential or current to convert it into heat [30,31]. However, the most common way in engineering is preheating the reactant gas or the liquid coolant in the thermal management system to warm up the PEMFC stack [32,33,34].

As the specific heat for water is almost four times higher than that of air, the liquid cooling is more suitable for the heat dissipation of high power PEMFCs compared with air cooling [35,36]. By that analogy, if the heat transfer fluid with equivalent specific heat capacity can be prepared in a certain temperature range, it can replace water to achieve better heat management of the PEMFC system. The microencapsulated phase change suspension (MPCS) corresponds exactly with this requirement. The microencapsulated phase change material (MPCM) has a typical core-shell structure that covers a micro-sized phase change material (PCM) with polymer materials [37,38]. Dispersing the MPCMs into single-phase fluid forms a new type of working substance called MPCS that can enhance heat transfer. This is because the latent heat generated by PCM during the phase transition of microcapsules can be converted into apparent specific heat capacity through the equivalent specific heat model. Meanwhile, the micro-convection effect between the microcapsules and carrier fluid will also increase the convective heat-transfer coefficient [39,40,41]. Therefore, the heat absorption / release and heat storage characteristics of MPCS have been widely used in temperature regulation of building, solar energy storage, photovoltaic/thermal system, heat exchanger design and many other fields [42,43].

**Figure 1:**
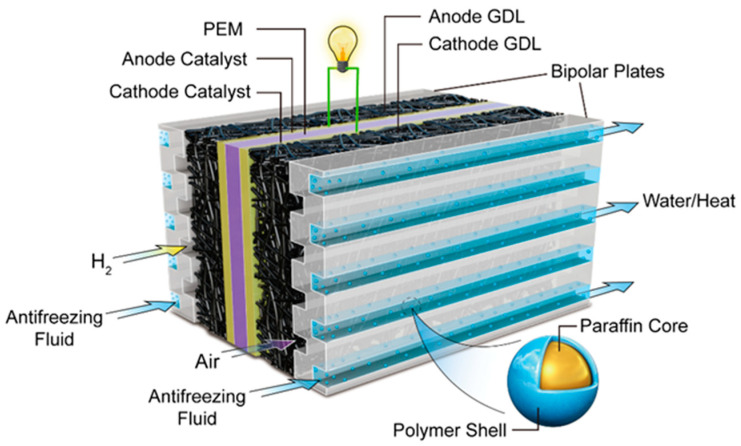
The mechanism diagram of phase change microcapsules for PEMFC cold start.

In this work, on the basis of previous research [44], an efficient thermal management technology of PEMFCs based on MPCS is proposed to improve the cold startup situation as shown in Figure 1. We designed and synthesized a kind of MPCM, the solidification heat release interval which was selected according to the cold start temperature of PEMFC; the end solidification temperature should be between 0 °C and cold start temperature. Its thermophysical and chemical properties, such as the morphology and particle size, chemical composition, phase transition characteristics, rheological behavior and temperature-regulated property were measured by experiments. Moreover, a PEMFC test platform for cold start with a thermal management system was established to compare the heat storage and release performance of MPCS with commercial antifreeze fluid using cold start experiments.

## 2. Experimental

### 2.1. Preparation of MPCS

In view of the advantages of paraffin, such as large latent heat, wide phase change temperature, multi-category, stability, nontoxicity and no corrosion [45], we chose paraffin as the core material in this paper, and a blend of polyacrylate and polyurethane as the shell material. The raw materials in the preparing process included paraffin OP0E from the Rubitherm Gmbh (Berlin, Germany), acrylate monomers and modified additives (polyurethane prepolymer, AppliChem GmbH, Darmstadt, Germany), styrene-maleic anhydride copolymer (SMA), polyvinyl alcohol (PVA) and glycol liquid (Aladdin, Shanghai, China), which can be used directly without further purification. As shown in Table 1, the solidification range of OP0E is −1~0 °C, and the heat storage capacity is 225 kJ/kg, which meet the application conditions of PEMFC cold startup. The typical preparing process of MPCS by in-situ polymerization has been reported in our previous work [44].

### 2.2. Characterization

The Scanning Electron Microscope (SEM) instrument (JSM-7001F, JEOL, Tokyo, Japan) and Malvern Mastersizer 2000 particle size analyzer (Malvern Instruments GmbH, Malvern, UK) were used to observe the surface morphology and particle size distribution of MPCMs. The molecular structures of the samples were analyzed by a Bruker Vertex-80 FTIR spectrometer (Bruker GmbH, Karlsruhe, Germany). The thermal storage performance parameters such as phase change temperature and latent heat of MPCM were measured with a TA Q2000 differential scanning calorimeter (DSC) instrument (TA Instruments GmbH, New Castle, PA, USA). The endothermic and exothermic cycles of samples greater than 4 mg were carried out between −30 °C and 30 °C at the scanning rate of 3.0 °C/min, which using standard alumina crucibles with a purge gas flow of 60 mL/min in nitrogen atmosphere. A MCR 301 Rotational Rheometer from Anton Paar (Graz, Austria) was used to determine the rheological properties of MPCS at different temperature and concentrations. The characteristic curves of heating and cooling processes of MPCS with different mass concentrations were performed using a R300SR-D thermal infrared imager of AVIO (Tokyo, Japan).

### 2.3. Test System and Measurement Process

**Figure 2:**
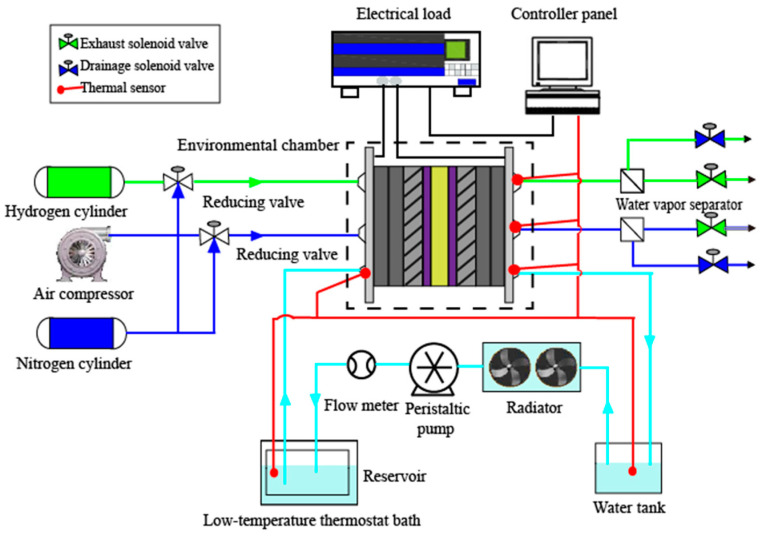
The schematic of the PEMFC platform for cold start with a thermal management system.

**Figure 3:**
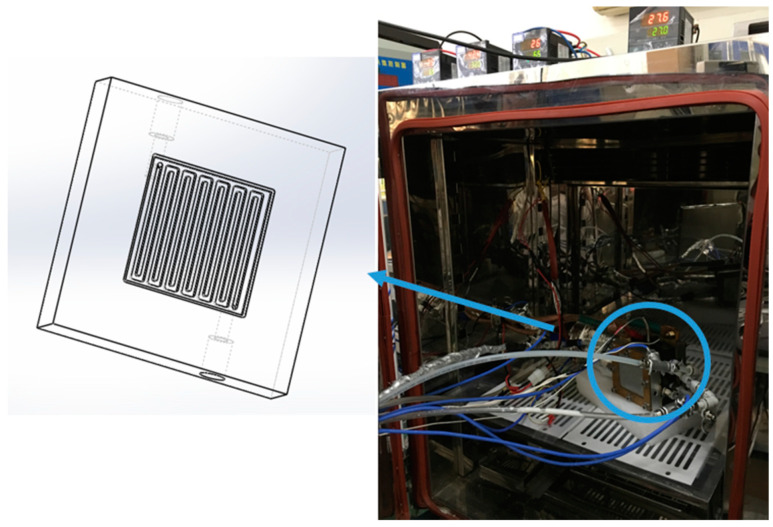
The physical map of the cold start experiment and the channel design of antifreeze fluid in bipolar plates.

The schematic diagram of PEMFC experimental system for cold start is shown in Figure 2, including temperature/humidity chamber, PEMFC performance testing platform, data acquisition system, thermal management system and specially designed PEMFC. The PEMFC performance testing platform consists of air and hydrogen supply system, nitrogen purge system, gas humidification system and control system. As for the thermal management system, it is made up of a cryogenic thermostatic bath, a reservoir, a peristaltic pump, a flow pulsation suppressor and a Coriolis mass flowmeter, which is used as an auxiliary heat source to preheat antifreeze fluid in the cold start experiments. The temperature testing points include the PEMFC temperature, the initial temperature, inlet and outlet temperature of the antifreeze fluid. The active area of PEMFC used in the experiments was 25 cm^2^ with Pt catalyst loading of 0.48 mg cm^−2^ in both the cathode and anode sides, and the proton exchange membrane and carbon paper were Nafion^®^211(Dupont, Wilmington, DE, USA) and SGL25BC (SGL Group, Wiesbaden, Germany). In order to approximate the heat transfer state of single cell in PEMFC stack, the serpentine coolant channel was machined on the back of bipolar plates with a length of 47.2 mm, a depth of 2 mm, a width of 2 mm, and separated by 1.6 mm lands. It was sealed by a sealing groove with a width of 1mm as shown in Figure 3.

Firstly, the activated polarization curves and AC impedance of the PEMFC were tested at room temperature under the following conditions: the PEMFC temperature was kept at 75 °C with an operating pressure of 1 atm. The relative humidity of cathode and anode reactant gases were 80% and the stoichiometric ratio of hydrogen and air was 1.5/3. Secondly, the dry nitrogen gas was used to purge the residual water at a flow rate of 1.2 L/min and 3 L/min in anode and cathode for 30 min until the gas humidity at the exit was less than 5%. Afterwards, the dried PEMFC was placed in the temperature/humidity chamber and cooled to the set temperature, then left to set for at least 3 h [46]. Then the cold start experiments were carried out under galvanostatic mode with the relative humidity of 0 and the flow rate of 0.6 L/min and 2.5 L/min for hydrogen and air. Finally, when the PEMFC reverted back to room temperature, it was reactivated and analyzed based on the measurement of polarization curves and AC impedance.

## 3. Results and Discussion

### 3.1. Morphology and Particle Size Distribution

**Figure 4:**
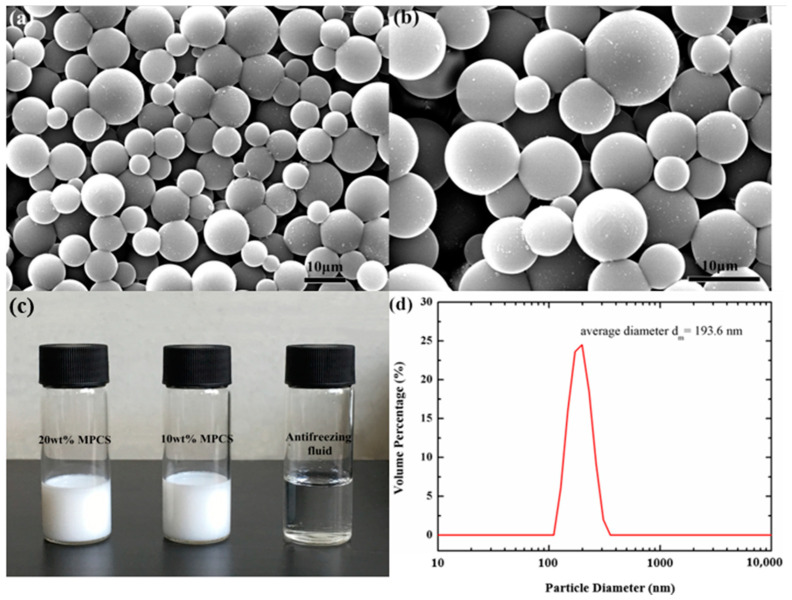
The SEM images of 1 wt% MPCS under different magnification: (**a**) 1:1300 (**b**) 1:2200. The photographs of (**c**) three liquids and particle size distribution of (**d**) 10 wt% MPCS.

As is shown in Figure 4c, the MPCS appears as milky emulsion which has good fluidity and the potential to be a heat transfer medium. The carrier fluid suitable for low temperature is composed of deionized water and glycol solution, and the ratio of the two can be adjusted according to the freezing point. The surface morphology of MPCMs can be observed by SEM; as displayed in Figure 4a,b, the MPCMs are spherical, smooth and dense on the surface as well as relatively uniform in size. There is almost no shell damage or agglomeration between capsules. The mechanical strength of the microcapsule shell is enough high to resist the volume shrinkage caused by the density variation between the reactive monomers and the polymer of the shell material and the variation between the solid and liquid density of the core material, so that there is no obvious collapse and crease in the surface of MPCM. Figure 4d exhibits the particle size distribution of 10 wt% MPCS; it can be perceived that the average size of the MPCMs shows unimodal distribution in the range of 100~400 nm and the peak value appears at 193.6 nm. The smaller the particle size, the larger the heat transfer area between the solid and the liquid [41,44]. It indicates that the prepared microcapsules are nanoscale, which is conducive to improving the heat transfer rate.

### 3.2. Chemical Composition

**Figure 5:**
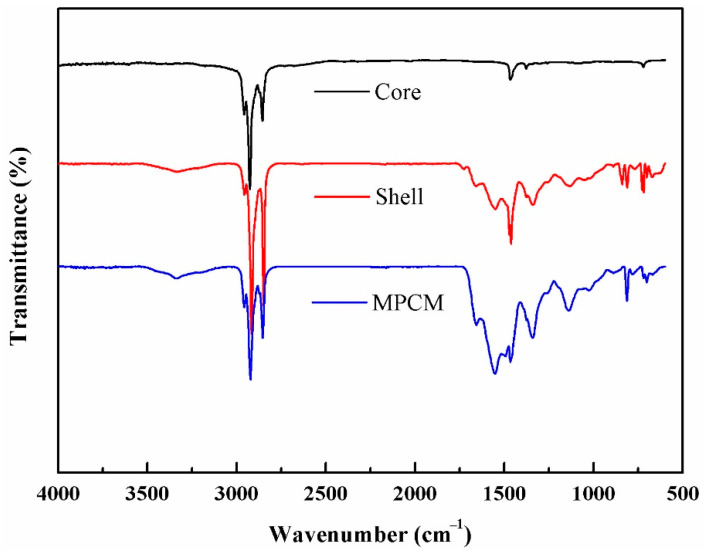
FT−IR spectra of core, shell and MPCM.

The chemical components of the core material, shell material and MPCM are shown as Figure 5. The absorption peaks at 2956 cm^−1^, 2916 cm^−1^ and 2848 cm^−1^ correspond to C–H stretching vibration of –CH_3_ and –CH_2_–. The signals at 1730 cm^−1^, 1550 cm^−1^ and 1131 cm^−1^ are separately assigned to C=O stretching vibration, N–H bending vibration and C–O–C stretching vibration absorption. Peaks appearing at 1252 cm^−1^ and 1023 cm^−1^ are responsible for C–O–C asymmetrical stretching vibration and symmetrical stretching vibration absorption. The absorption peaks at 1462 cm^−1^, 1374 cm^−1^, 835 cm^−1^ and 701 cm^−1^ are associated with –CH_2_– bnding vibration, –CH_3_ symmetrical deformation vibration and rocking vibration, and the characteristic peak of the benzene ring. The uncharacteristic signals above indicate that the polymer shell is formed by a blend of polyacrylate and polyurethane. In the spectrum of core material, peaks occurred at 2956 cm^−1^, 2916 cm^−1^ and 2848 cm^−1^, which are due to C–H stretching vibration of –CH_3_ and –CH_2_–, the peaks at 1472 cm^−1^ and 1377 cm^−1^ present –CH_3_ asymmetrical bending vibration and symmetrical bending vibration and the absorption peak near 719 cm^−1^ belongs to thr –CH_2_– rocking vibration, which are all characteristics for paraffin. The characteristic absorption peaks of the prepared MPCM are completely corresponding to the core material and shell material, indicating that the paraffin OP0E has been successfully coated by the blend of polyacrylate and polyurethane.

### 3.3. Phase Change Temperature and Enthalpy

**Figure 6:**
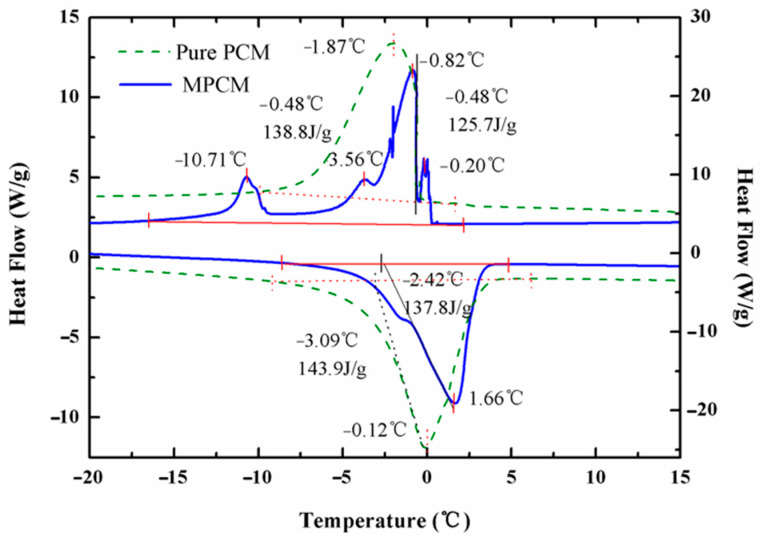
The DSC curves of MPCM and pure PCM during phase transition.

**Figure 7:**
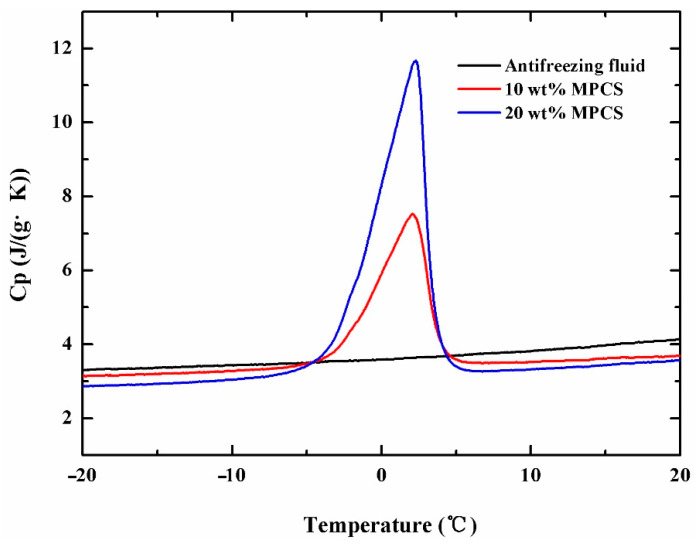
The specific heat capacity with different latex concentration.

The thermal analysis was performed on the dry MPCM and pure PCM as shown in Figure 6. The DSC curve of the MPCM indicates the onset melting temperature and end temperature are −2.42 °C and 4.84 °C with a peak value of 1.66 °C. According to the crystallization curve, the onset solidification temperature is −0.48 °C, the peak value is −0.82 °C, and the end temperature is −15.4 °C. The latent heat of crystallization for MPCM is very considerable, about 125.7 J/g. Compared with the onset melting temperature in the heating process, the onset solidification temperature during the cooling process has the phenomenon of supercooling, which is about 3.94 °C, much lower than the previous reports [47,48]. In addition, the influence of polymer shell with low thermal conductivity still exists, the onset melting temperature and peak value of MPCM increase slightly relative to pure PCM, while the onset solidification temperature and peak value of MPCM decrease slightly, as well as the latent heat. The encapsulation ratio of the MPCM which reflects the mass fraction of pure core material in MPCM is calculated by formula below [49]:

(1) $$\eta \;\left(\%\right)=\frac{\Delta H_{MPCM}}{\Delta H_{PCM}}\;\times 100\%$$

where Δ*H_MPCM_* and Δ*H_PCM_* represent the transformation enthalpy of MPCM and pure PCM, thus, the encapsulation ratio of the MPCM prepared is 87.4%. The unique characteristics of the phase transition process can be reflected by the measurement of specific heat capacity, the results are shown in Figure 7. The apparent specific heat capacity of MPCS at different mass concentrations increase rapidly in the phase change temperature range of paraffin, compared to the commercial antifreezing fluid. The maximum specific heat capacities of 20 wt% and 10 wt% MPCS are 11.68 J g^−1^ K^−1^ and 7.53 J g^−1^ K^−1^, much larger than that of antifreezing fluid. It indicates that the exothermicity of the suspension is improved significantly, the phase enthalpy of MPCS enlarges with the increase of its mass concentration.

### 3.4. Rheological Behavior

**Figure 8:**
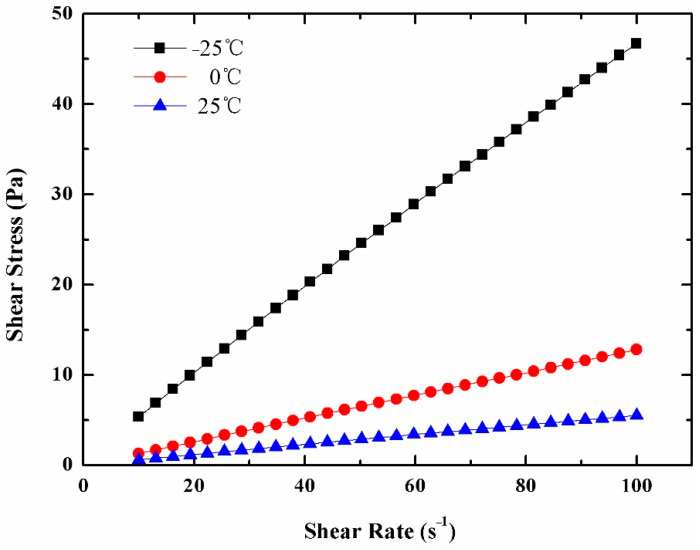
The relationship between shear stress and shear rate of 25 % MPCS at −25 °C, 0 °C and 25 °C.

**Figure 9:**
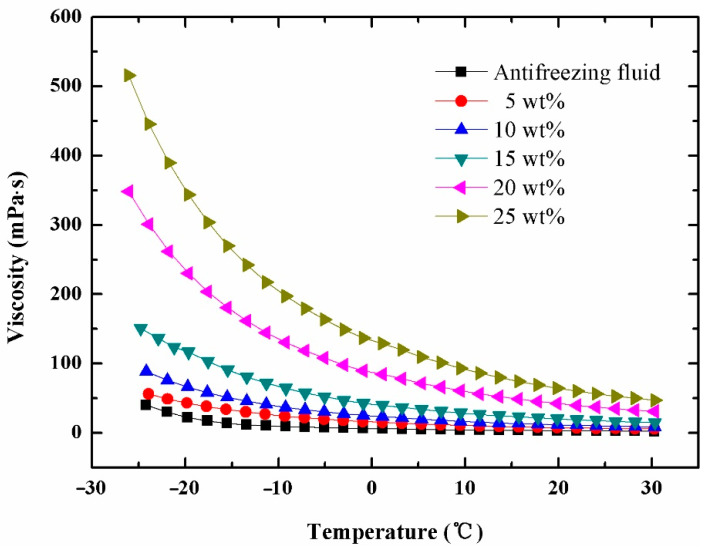
The relationship between viscosity and temperature at different mass concentrations.

Figure 8 shows the corresponding relation of shear stress and shear rate less than 100 s^−1^ of 25 wt% MPCS at −25 °C, 0 °C and 25 °C. All three curves pass through the origin and increase linearly as well as monotonically, exhibiting the Newtonian fluid characteristics when the liquidity index m=1. Therefore, the MPCS with mass concentrations below 25 wt% can be regarded as Newtonian fluid. According to Figure 9, with the increase of temperature the kinematic viscosity reduces gradually under the same concentration of suspension, and the differences in kinematic viscosity between suspensions with different concentrations decrease gradually. When PEMFC operates in cold conditions, such as at −10 °C, the kinematic viscosity of 5 wt%, 10 wt%, 15 wt%, 20 wt% and 25 wt% MPCS are 24.18, 36.73, 64.22, 130.72 and 197.22 mPa s. Taking 9.3 mPa s of commercial antifreeze fluid as a reference, it shows that the kinematic viscosity of MPCS with mass concentrations below 25 wt% is in the acceptable range, so that the prepared MPCS is an ideal choice for PEMFC antifreeze fluid. 

### 3.5. Thermal Imaging

**Figure 10:**
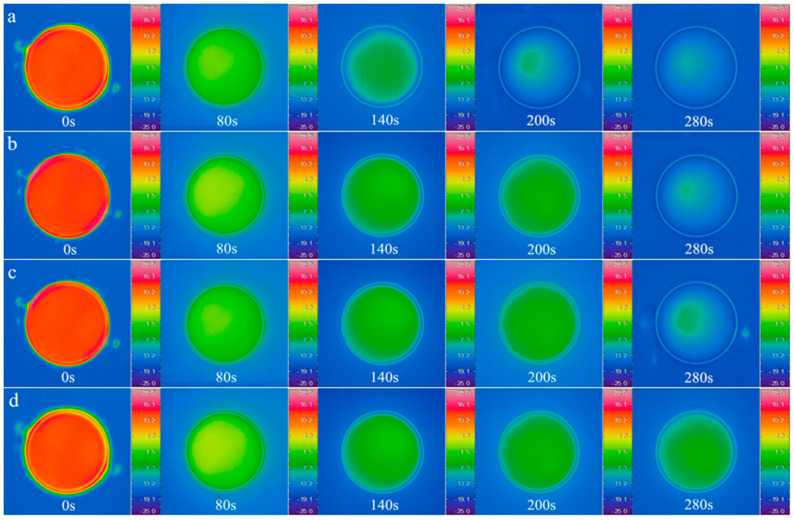
Infrared camera images of petri dishes with antifreeze fluid and different mass concentrations of MPCS under cooling process: (**a**) antifreeze fluid (**b**) 10 wt % (**c**) 20 wt % (**d**) 30 wt %.

**Figure 11:**
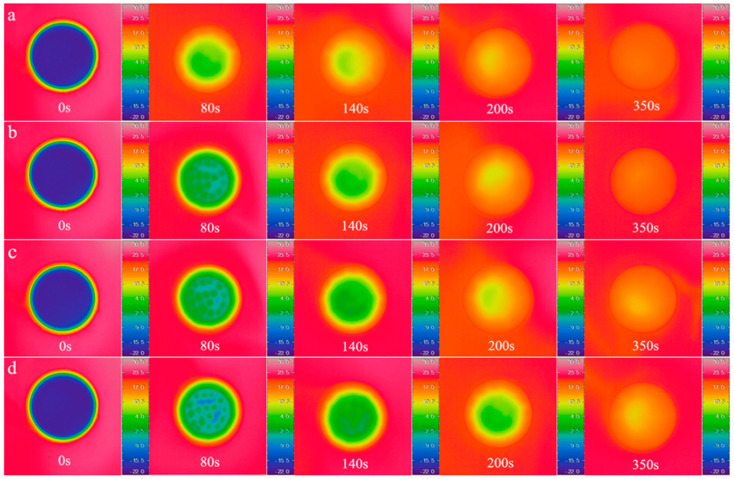
Infrared camera images of petri dishes with antifreeze fluid and different mass concentrations of MPCS under heating process: (**a**) antifreeze fluid (**b**) 10 wt % (**c**) 20 wt % (**d**) 30 wt %.

The initial temperature of the samples in the petri dishes was kept at 15 °C, and the temperature of cryogenic thermostatic bath was set at −15 °C. The temperature field of antifreeze fluid, 10 wt%, 20 wt% and 30 wt% MPCS were recorded by infrared thermal imager over time, and the timer started when the samples were put into the thermostat water bath. The infrared images at 0 s, 80 s, 140 s, 200 s and 280 s were selected for analysis as shown in Figure 10, and the initial temperature of the four groups were the same at 0 s. At 140 s, the samples in the petri dishes reached the phase transition temperature of the microcapsules, so that the temperature of commercial antifreeze fluid was significantly lower than that of the three MPCS samples. At 200 s, the three MPCS samples were in the phase transition interval, and the commercial antifreeze fluid continued to cool down. At 280 s, only the phase transition of 30 wt% MPCS was not over, and the other three groups were close to the temperature equilibrium state. In the heating experiment, the cryogenic thermostatic bath was used to cool the samples in the petri dishes to −20 °C, and the thermostat water bath was set at 20 °C. The infrared images at 0 s, 80 s, 140 s, 200 s and 350 s were selected for analysis as shown in Figure 11. Compared with the commercial antifreeze fluid, the temperature variations of all MPCS samples were slower, and the higher the mass concentration of the MPCMs during heating, the greater the effect of delay on heat accumulation.

**Figure 12:**
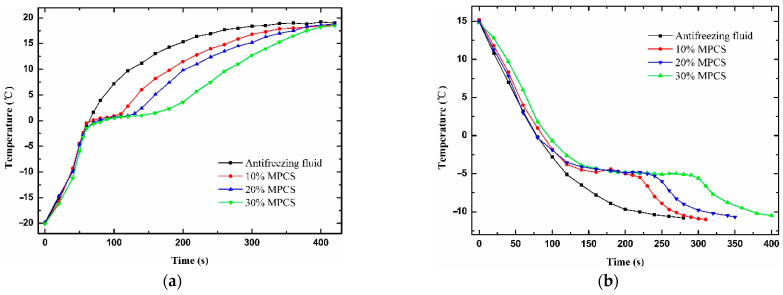
Temperature of antifreeze fluid and different mass concentrations of MPCS under (**a**) heating and (**b**) cooling process.

From the heating and cooling characteristic curves of commercial antifreeze fluid and MPCS at different mass concentrations in Figure 12, it is observed that the developing trends of the four curves are different when the temperature reaches the onset melting temperature of microcapsules in the process of heating. At the same time, the corresponding temperature of commercial antifreeze fluid is the highest, and that of MPCS becomes lower as the mass concentration increases. On the contrary, the corresponding temperature becomes higher as the mass concentration of MPCS increases in the cooling process. Meanwhile, the plateaus of MPCS heating and cooling curves occur at around 0 °C and −4 °C, respectively, indicating the temperature-regulated property of the MPCMs. In addition, the content of MPCMs determines the time of constant temperature. These results show that the prepared MPCS has application potential in cold start-up of PEMFCs.

### 3.6. Cold Start Characteristics

During the cold start experiments, the ambient temperatures set by the temperature/humidity chamber were −10 °C, −15 °C and −20 °C, respectively. The commercial antifreeze fluid, 10 wt% and 20 wt% MPCS, were pumped into the thermal management circulating system to heat the PEMFC. The inlet temperature of the three liquids was constant at 12 °C, the inlet flow rate was 0.029 L/min and the current density loaded in the start-up process of galvanostatic mode was 0.04 A cm^−2^ with a relative humidity of 0 and a flow rate of 0.6 L/min and 2.5 L/min for hydrogen and air.

**Figure 13:**
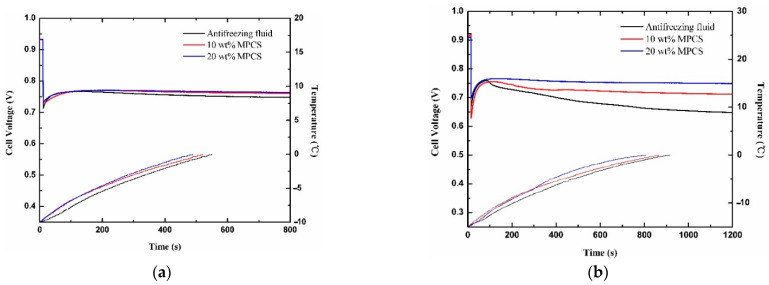

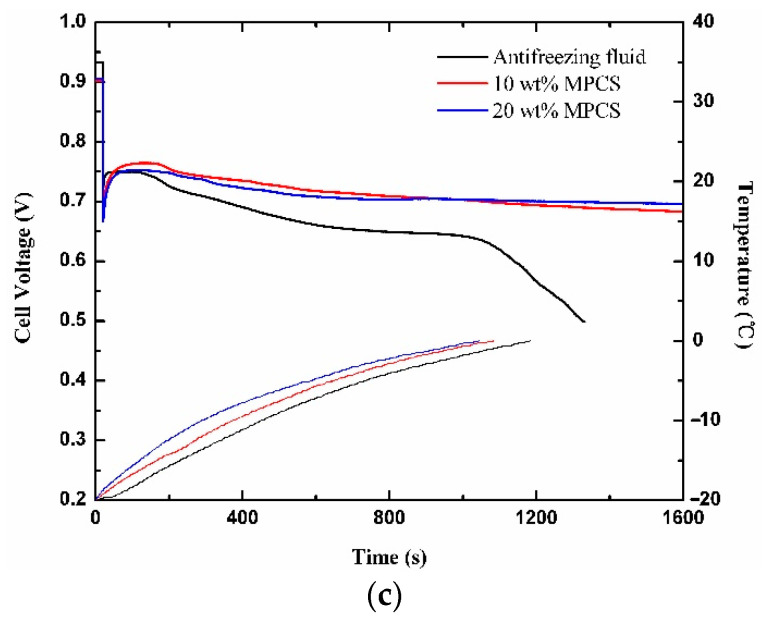
The cell voltages and temperatures at different mass concentrations of antifreeze fluid (**a**) −10 °C (**b**) −15 °C (**c**) −20 °C.

Figure 13 exhibits the variation curves of cell voltage and temperature with time under the heating of antifreeze fluid with different mass concentrations at cold start operations. All the voltage curves first rush down rapidly after loading, then recover slowly and maintain or gradually decline. When the ambient temperature is −10 °C, the cell voltages under the heating of 20 wt% MPCS, 10 wt% MPCS and commercial antifreeze fluid remain almost constant, fall by 0.007 V, 0.01 V and 0.019 V at 800 s, respectively. The temperature of PEMFC rises from −10 °C to 0 °C in 489 s, 521 s and 550 s. While the ambient temperature is −15 °C, the decreasing slopes of cell voltage under the heating of 20 wt% MPCS, 10 wt% MPCS and commercial antifreeze fluid increase successively. The difference of output performance tends to be obvious, and the corresponding voltage drops are 0.017 V, 0.043 V and 0.111 V. It takes more time for the PEMFC temperature to rise from −15 °C to 0 °C, about 807 s, 867 s and 914 s. As for when the ambient temperature is −20 °C, the PEMFC voltage under the heating of 20 wt% and 10 wt% MPCS decrease by 0.056 V and 0.082 V at 1600 s. However, the cell voltage under commercial antifreeze fluid heating drops swiftly at 1000 s, so that the test has to be stopped. It takes 1044 s, 1083 s and 1183 s for the PEMFC to rise to 0 °C. The results show that the PEMFC under the heating of 20 wt% MPCS has the best output performance and shortest start-up time, followed by the performance of PEMFC under 10 wt% MPCS heating, which is better than the PEMFC under the heating of commercial antifreeze fluid.

The key to determining whether the cold start will be successful or not is the relationship between ice accumulation rate and heating rate in PEMFC. The heat absorbed by the PEMFC comes from internal heat source and auxiliary heat source. The heat generated in a PEMFC includes entropic heat of reactions, irreversibilities of the electrochemical reactions and ohmic resistances, as well as water condensation [50,51]. The PEMFC operates under the same conditions in each experiment, so that its internal heat source produces the same amount of heat. For the three kinds of antifreeze fluid, the addition of MPCMs can transform the latent heat generated during the phase transition into apparent specific heat capacity. That is, the total apparent specific heat capacity of the MPCS enlarges with the increase of the mass concentration for MPCMs. Combined with the micro-convection effect, the convective heat transfer coefficient of the MPCS will increase. As a consequence, the higher the mass concentration of MPCS, the more heat it provides to the PEMFC, and the greater the possibility of successful cold start.

### 3.7. Performance before and after Cold Start

**Figure 14:**
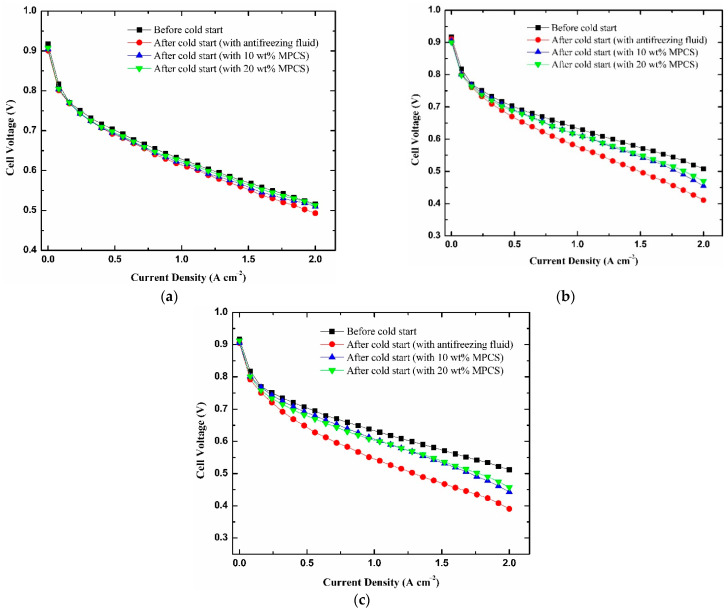
The polarization curves of PEMFC before and after cold starts at different mass concentrations of antifreeze fluid (**a**) −10 °C (**b**) −15 °C (**c**) −20 °C.

In Figure 14, the polarization curves of PEMFC before and after cold start experiments under the heating of antifreeze fluid with different mass concentrations were tested to characterize the effect of MEA degradation on PEMFC performance. It is not difficult to find that compared with the initial polarization curves before cold start, the PEMFC performances after cold start decrease in varying degrees and the decline range is related to the output performance of PEMFC in the process of cold start. When the ambient temperature is −10 °C, the polarization curves of the PEMFC heated by 20 wt% MPCS, 10 wt% MPCS and commercial antifreeze fluid have a small gap between before cold start. At 2.0 A cm^−2^, the cell voltage before cold start is 0.516 V, the cell performance under the heating of 20 wt% MPCS, 10 wt% MPCS and commercial antifreeze fluid decrease by 0.6%, 1.2% and 4.4%, respectively. While the ambient temperature is −15 °C, the cell voltages under the above three antifreeze fluid heatings fall by 7.5%, 10.4% and 19.1% compared with those before cold start at 2.0 A cm^−2^, and the declines are larger than those at −10 °C. As for when the ambient temperature is −20 °C, the cell voltage under commercial antifreeze fluid heating has fallen below 0.4 V at 2.0 A cm^−2^, the performance of PEMFC under the above three antifreeze fluid heating reduces by 10.7%, 13.5% and 23.7% compared with those before cold start. The declines increase further than those at −15 °C.

During the cold start, the blocking and deformation of three-phase channels in MEA, the interface delamination of CL from membrane and GDL, rough cracks and pinhole formations on the membrane surface, the detachment of PTFE particles on carbon paper and fracture of carbon fibers are all caused by freezing, leading to the performance degradation of PEMFC [52,53,54]. The experiment results indicate that the lower the ambient temperature is, the more serious the damage of PEMFC is, and the more obvious the output performance degradation is. In addition, the higher the mass concentration of MPCS, the smaller the degradation amplitude of PEMFC performance after cold start, and the smaller the damage to PEMFC components during cold start.

## 4. Conclusions

In this paper, we designed and synthesized MPCMs which can be used to solve the problem of PEMFC cold start by in-situ polymerization. The FT-IR analysis shows that paraffin OP0E has been successfully coated by the blend of polyacrylate and polyurethane. SEM and PSD indicate that the MPCM was spherical in shape, smooth and dense in surface and well coated. The particle size distribution ranges from 80 to 400 nm. The DSC results reveal a crystallization enthalpy of 125.7 J/g, and encapsulation ratio of 87.4 % to the MPCMs with undercooling of 5.32 °C. The apparent specific heat capacity of MPCS enlarges with the increase of its mass concentration, so that the phase enthalpy will increase. The rheological properties indicate that the suspensions of 25% and below can be used as Newtonian fluid and the kinematic viscosity of suspensions up to 25% is within acceptable range. The thermal imaging analysis shows that the samples containing MPCMs have good temperature regulation performance compared with commercial antifreeze fluid. On this basis, a PEMFC platform for cold start with a thermal management system was established to compare the exothermic performance of MPCS and commercial antifreeze fluid. The results show that when the ambient temperatures were −10 °C, −15 °C and −20 °C, the PEMFC under the heating of 20 wt% MPCS had the best output performance and shortest start-up time, followed by the performance of PEMFC under 10 wt% MPCS heating, which is better than the PEMFC under the heating of commercial antifreeze fluid. The variation of polarization curves before and after cold start show that the higher the mass concentration of MPCS, the smaller the degradation amplitude of PEMFC performance after cold start, and the smaller the damage to PEMFC components during cold start. All of these prove that the MPCS has a stronger heat transfer capacity than the commercial antifreeze fluid, leading to a better solution for the problem of PEMFC cold start.

## Figures and Tables

**Table 1:** The performance parameters of OP0E.

Parameter	Value
Temperature range of melting (°C)	−1~2
Temperature range of solidification (°C)	−1~0
Heat storage capacity (kJ/kg)	225
Specific heat (kJ/(kg K))	2
Density solid at 15 °C (kg/L)	0.88
Density liquid at 80 °C (kg/L)	0.77
Volume expansion with phase change (%)	12.5
Thermal conductivity coefficient (W/(m K))	0.2

## Data Availability

The data presented in this study are available in this article.
